# A study of ECT on 278 children and adolescents; methodological, conceptual, and ethical concerns

**DOI:** 10.1002/brb3.2866

**Published:** 2022-12-26

**Authors:** John Read, Colin Ross, Sami Timimi

**Affiliations:** ^1^ School of Psychology University of East London London UK; ^2^ Colin A. Ross Institute for Psychological Trauma Dallas Texas USA; ^3^ Lincolnshire Partnership NHS Foundation Trust Lincolnshire UK

**Keywords:** adolescents, adverse effects, electroconvulsive therapy, memory loss, ethics, polypharmacy

A study of ECT on 278 suicidal children and adolescents aged 12–17 (Chen et al., 2022) reports a 52% response rate and an ‘‘acceptable’’ level of adverse effects. However, the paper contains serious methodological flaws and ignores important conceptual and ethical issues.

## 1 METHODOLOGICAL PROBLEMS

All the participants were also receiving antidepressants. Many were also taking antipsychotics (46%), and some were on mood stabilizers (11%). How many were on benzodiazepines is unclear. This is reported as ‘‘most’’ in the abstract, and 23% in table 1 (Chen et al., 2022); and the most frequently prescribed benzodiazepine was reportedly being taken by 29% of the participants. Since participants were on between one and four psychiatric drugs, little can be concluded about which, if any, of the five interventions (four drugs plus ECT) were responsible for outcomes. The authors, however, attributed the reported improvements entirely to ECT.

The authors do not state when the antidepressants were started. If they were started about the same time as the ECT (which seems likely given the participants' relatively high pre‐ECT depression scores), it would be impossible to tell how much of the response was due to ECT. The results cannot be interpreted as an effect of ECT without this information.

There was no placebo or control group; therefore, the contribution of placebo to the responses cannot be determined. The high levels of care and attention, and strong expectations, involved with ECT produce substantial placebo effects (Rasmussen, 2009; Read et al., 2019; Ross, 2006).

The evaluation of side effects has several methodological limitations. Memory loss was estimated by ‘‘interview’’ and ‘‘observation,’’ not by standardized tests of cognitive functioning (Robertson & Pryor, 2006). It is not stated how long after ECT memory was evaluated. It is not reported whether any evaluation of memory occurred prior to ECT, without which any evaluations after ECT are hard to interpret. The interviews estimating memory loss were conducted by the ‘‘psychiatrist who applied ECT’’ rather than by an independent rater.

The Clinical Global Impression scale (a one‐item subjective assessment of degree of unwellness) was also completed by the treating psychiatrists. It is not stated whether the treating psychiatrists also completed the Hamilton Depression Scale. There was no mention in the Limitations section of the need for independent raters of treatment response and adverse effects.

## 2 CONCEPTUAL PROBLEMS

No information is provided about the participants’ life histories or current circumstances. There is no reference to any trauma or adverse childhood experiences. No mention is made of any prior or ongoing treatment efforts to address these potential causes of the participants’ depression, such as individual or family psychotherapy. The authors’ decontextualized approach is consistent with no comment being made about the fact that 85% of the participants were girls. This is a highly skewed sample requiring explanation.

ECT is described by Chen et al. as ‘‘highly effective’’ for the treatment of depression. There have, however, been no placebo‐controlled studies of ECT for depression since 1985, and all 11 studies prior to that date were very small, severely flawed, and conducted on adults (Read & Bentall, 2010; Read et al., 2019). There have been no placebo‐controlled studies on children or adolescents.

## 3 ETHICAL PROBLEMS

The use of ECT on adults is controversial (Meechan et al., 2021; Read & Moncrieff, 2022; Read et al., 2018), in part because persistent or permanent memory loss occurs in between 12% (Sackeim et al., 2007) and 55% (Rose et al., 2003) of recipients. Its use on 278 children and adolescents, whose brains were still developing, seems particularly problematic, especially in the absence of any placebo‐controlled studies in that age group.

Similarly, it is of concern that all these depressed, suicidal teenagers were given antidepressants. In 2004, the Food and Drug Administration issued a Black Box warning that antidepressants *increase* the risk of suicidality in children and adolescents This has recently been confirmed as being ‘‘firmly rooted in solid data’’ (Spielmans et al., 2020). Government guidelines in the United Kingdom state that, ‘‘A child or young person prescribed an antidepressant’’ (which must only happen ‘‘in combination with concurrent psychological intervention’’) … ‘‘should be closely monitored for the appearance of suicidal behaviour’’ (NICE, 2019).

It is of equal concern that nearly half of these young people were also on antipsychotics, which have serious adverse effects in adults, especially since none had a psychosis‐related diagnosis (table 1 inChen et al., 2022). When one considers the fact that ‘‘most’’ of the participants were also on benzodiazepines, these young people were receiving a high level of polypharmacy.

We n ote that 9% of the participants had “cardiovascular disorders.” None of these 25 participants were excluded from the study, despite cardiovascular failure being the leading cause of ECT‐related deaths (Lindblad et al., 2023; Read et al., 2019).

Readers are not told how many of the children and adolescents who were asked to participate agreed, and how many declined. That raises questions about whether they genuinely had a choice and, if they did, was it a genuinely informed choice? Readers are left wondering what they, and their caregivers, were told about ECT and its various potential adverse effects. Furthermore, we are not told what happened if the children and adolescents declined but their caregivers agreed?

Despite the potential minimizing bias of having the administrators of a treatment estimate its adverse effects, 68% of the participants were reported to have suffered memory problems as a result of the ECT. Furthermore, 34% of the participants were reported to experience delirium. This was interpreted as evidence that the level of adverse effects from ECT for children and adolescents is “acceptable” and that ECT is “a safe choice”. We disagree.

## 4 CONCLUSION

Conducting research on psychiatric treatments is always a good idea, and the authors are to be commended for their efforts. However, there are so many methodological flaws and conceptual problems that no conclusions can be drawn about the contribution of ECT to the results. We hope that the paper will not be cited as providing evidence that ECT is ‘‘safe and effective’’ for children and adolescents. The paper does not demonstrate that that is the case. It does raise ethical concerns about the well‐being of the children and adolescents involved.

## CONFLICTS OF INTEREST

John Read has been a paid Expert Witness in two ECT legal cases, in the USA and Canada. The other authors declare no conflict of interest.

### PEER REVIEW

The peer review history for this article is available at https://publons.com/publon/10.1002/brb3.2866.

## References

[brb32866-bib-0001] Chen, X. , Fu, Y. , Zou, Q. , Zhang, Y. , Qin, X. , Tian, Y. , Yan, Y. , Chen, Q. , Zou, L. , Zhao, B. , & Li, X. (2022). A retrospective case series of electroconvulsive therapy in the management of depression and suicidal symptoms in adolescents. Brain and Behavior, 12, e2795. 10.1002/brb3.2795 36259943 PMC9660487

[brb32866-bib-0002] Lindblad, L. , Nordenskjöld, A. , Otterbeck, A. , & Nordenskjöld, A. (2023). Risk factors for mortality of medical causes within 30 days of electroconvulsive therapy. Journal of Affective Disorders, 320, 527–533. 10.1016/j.jad.2022.10.008 36209782

[brb32866-bib-0003] Meechan, C. , Laws, K. , Young, A. , McLoughlin, D. , & Jauhar, S. (2021). A critique of narrative reviews of the evidence‐base for ECT in depression. Epidemiology and Psychiatric Sciences, 31, e10. 10.1017/S2045796021000731 PMC8851059 35083968

[brb32866-bib-0004] National Institute for Health and Care Excellence . (2019). Depression in children and young people: Identification and management. NICE guideline NG134. London: NICE. https://www.nice.org.uk/guidance/ng134/chapter/recommendations 31577402

[brb32866-bib-0015] Rasmussen, K. (2009). Sham electroconvulsive therapy studies in depressive illness: A review of the literature and consideration of the placebo phenomenon in electroconvulsive therapy practice. Journal of ECT, 25, 54–59. 10.1097/YCT.0b013e3181719b23 18580816

[brb32866-bib-0005] Read, J. , & Bentall, R. (2010). The effectiveness of electroconvulsive therapy: A literature review. Epidemiology and Psychiatric Sciences, 19, 333–347. 10.1017/S1121189X00000671 21322506

[brb32866-bib-0016] Read, J. , Harrop, C. , Geekie, J. , & Renton, J. (2018). An audit of ECT in England 2011‐2015: Usage, demographics, and adherence to guidelines and legislation. Psychology and Psychotherapy: Theory, Research and Practice, 91, 263–277. 10.1111/papt.12160 29052308

[brb32866-bib-0006] Read, J. , Cunliffe, S. , Jauhar, S. , & Mcloughlin, D. (2019). Should we stop using electroconvulsive therapy? British Medical Journal, 364, k5233. 10.1136/bmj.k5233 30626580

[brb32866-bib-0007] Read, J. , Kirsch, I. , & Mcgrath, L. (2019). Electroconvulsive therapy for depression: A review of the quality of ECT vs sham ECT trials and meta‐analyses. Ethical Human Psychiatry and Psychology, 21, 64–103. 10.1891/EHPP-D-19-00014

[brb32866-bib-0008] Read, J. , & Moncrieff, J. (2022). Depression: Why drugs and electricity are not the answer. Psychological Medicine, 52, 1401–1410. 10.1017/S0033291721005031 35100527

[brb32866-bib-0009] Robertson, H. , & Pryor, R. (2006). Memory and cognitive effects of ECT: Informing and assessing patients. Advances in Psychiatric Treatment, 12, 228–238. 10.1192/apt.12.3.228

[brb32866-bib-0010] Rose, D. , Wykes, T. , Leese, J. , Bindmann, J. , & Fleischmann, P. (2003). Patients’ perspectives of electroconvulsive therapy: Systematic review. British Medical Journal, 326, 1363–1366. 10.1136/bmj.326.7403.1363 12816822 PMC162130

[brb32866-bib-0011] Ross, C. (2006). The sham ECT literature: Implications for consent to ECT. Ethical Human Psychology and Psychiatry, 8, 17–28. 10.1891/ehpp.8.1.17 16856307

[brb32866-bib-0012] Sackeim, H. , Prudic, J. , Fuller, R. , Keilp, J. , Lavori, P. , & Olfson, M. (2007). The cognitive effects of ECT in community settings. Neuropsychopharmocology, 32, 244–254. 10.1038/sj.npp.1301180 16936712

[brb32866-bib-0013] Spielmans, G. , Spense‐Sing, T. , & Parry, P. (2020). Duty to warn: Antidepressant black box suicidality warning is empirically justified. Frontiers in Psychiatry, 11, 18. 10.3389/fpsyt.2020.00018 32116839 PMC7031767

